# Helper T Cell (CD4^+^) Targeted Tacrolimus Delivery Mediates Precise Suppression of Allogeneic Humoral Immunity

**DOI:** 10.34133/2022/9794235

**Published:** 2022-07-16

**Authors:** Jia Shen, Chang Liu, Pengpeng Yan, Meifang Wang, Luying Guo, Shuaihui Liu, Jianghua Chen, Jessica M. Rosenholm, Hongfeng Huang, Rending Wang, Hongbo Zhang

**Affiliations:** ^1^Kidney Disease Center, The First Affiliated Hospital, College of Medicine, Zhejiang University; Key Laboratory of Kidney Disease Prevention and Control Technology, Zhejiang Province, China; ^2^Pharmaceutical Sciences Laboratory, Faculty of Science and Engineering, Åbo Akademi University, Turku 20520, Finland; ^3^Turku Bioscience Centre, University of Turku and Åbo Akademi University, Turku 20520, Finland; ^4^Organ Donation and Coordination Office, The First Affiliated Hospital, School of Medicine, Zhejiang University, Hangzhou 310003, China; ^5^Department of Orthopaedics, Shanghai Key Laboratory for Prevention and Treatment of Bone and Joint Diseases, Shanghai Institute of Traumatology and Orthopaedics, Ruijin Hospital, Shanghai Jiao Tong University School of Medicine, Shanghai 200025, China

## Abstract

Antibody-mediated rejection (ABMR) is a major cause of dysfunction and loss of transplanted kidney. The current treatments for ABMR involve nonspecific inhibition and clearance of T/B cells or plasma cells. However, the prognosis of patients following current treatment is poor. T follicular helper cells (Tfh) play an important role in allograft-specific antibodies secreting plasma cell (PC) development. Tfh cells are therefore considered to be important therapeutic targets for the treatment of antibody hypersecretion disorders, such as transplant rejection and autoimmune diseases. Tacrolimus (Tac), the primary immunosuppressant, prevents rejection by reducing T cell activation. However, its administration should be closely monitored to avoid serious side effects. In this study, we investigated whether Tac delivery to helper T (CD4^+^) cells using functionalized mesoporous nanoparticles can block Tfh cell differentiation after alloantigen exposure. Results showed that Tac delivery ameliorated humoral rejection injury in rodent kidney graft by suppressing Tfh cell development, PC, and donor-specific antibody (DSA) generation without causing severe side effects compared with delivery through the drug administration pathway. This study provides a promising therapeutic strategy for preventing humoral rejection in solid organ transplantation. The specific and controllable drug delivery avoids multiple disorder risks and side effects observed in currently used clinical approaches.

## 1. Introduction

Antibody-mediated rejection (ABMR), the leading cause of graft dysfunction in solid organ transplantation, impacts long-term graft and recipient survival [1]. Increased donor-specific antibodies (DSA) against graft-derived major-histocompatibility-complex (MHC) classes I and II antigens activate the complement system and the endothelium, resulting in histological injury of the allograft. Therefore, ABMR can be treated by clearing DSA through plasma exchange and antibody neutralization or by inhibiting DSA generation. Plasma cells (PC) are DSA-producing cells that differentiate from B cells. Various clinical trials have investigated PC and B cell inhibitions (bortezomib, atacicept, and belimumab) [2–4], as well as B cell depletion (rituximab, ofatumumab, ocrelizumab, and epratuzumab) [3–6]; however, the outcomes of these treatments have been poor, especially in refractory and later ABMR cases.

The generation of PC is a T cell-dependent B cell response [7, 8]. Briefly, alloantigen peptides are captured and delivered by recipient dendritic cells to naïve helper T cells (CD4^+^) in the secondary lymphoid organs, such as spleen and lymph nodes [9]. Activated naïve CD4^+^ T cells differentiate into T follicular helper (Tfh) cells and migrate towards the germinal center (GC), where they help the B cells to undergo GC selection. GC B cells ultimately develop into short-lived antibody secreting PC or long-lived PC and memory B cells. Proliferation of reactivated long-lived PC and memory B cells also depends on Tfh cytokine signals, especially IL-4 and IL-21, in GC reactions. IL-21R, which delivers the pleiotropic effects of IL-21, is widely expressed on multiple immune cells, including T, B, and plasma cells. As one of the effectors of GC reaction, IL-21R overexpresses during humoral rejection in kidney transplantation (Figure S1), suggesting the vital role of Tfhs in ABMR. ABMR symptoms can be reduced by Tfh cell monitoring [10, 11], clearance [10], and uncoupling of their interactions with B cells [12]. Preliminary results have also shown that splenectomy and splenic irradiation can be used as rescue therapies for refractory and severe ABMR [8, 13–16]. However, the limitations of these therapies have been noted, including increased surgical and infection risks. Tfhs mainly mature and distribute in secondary lymphoid organs, such as the spleen and draining lymph nodes, where alloantigens are presented [17]. Cellular- or locus-specific therapy approaches are rarely performed because the specific drug delivery to lymphoid tissues is restricted [18].

Tacrolimus (Tac), a macrolide calcineurin inhibitor, can inhibit T cell development signals by uncoupling the dephosphorylation of the nuclear factor of activated T cells. After approval by the US Food and Drug Administration (FDA) in 1994, it became the primary immunosuppressant drug in transplantation. Tfh cells are the CD4^+^ cell subsets that are most sensitive to Tac [19]. Thus, Tac is a suitable candidate drug for Tfh inhibition. The efficacies of Tac for Tfh inhibition have not been conclusively determined, and barriers to Tac delivery to optimal physical sites may account for the low levels of efficacy. Thus, the abilities of nanoparticles and other carriers in enhancing the delivery of these molecules to target sites need to be explored and evaluated. These approaches can promote ABMR therapy by mediating the accumulation of immunosuppressants in specific organs and within specific cellular compartments, as well as by inhibiting the differentiation of certain immune cell types, while suppressing the side effects induced by systemic immune inhibition.

Rationally designed nanomaterials can encapsulate payloads, such as drugs and biomolecules and facilitate their targeted delivery to treat various diseases [20–24]. We hypothesize that this technology is also applicable for delivery of immunosuppressive drugs in organ transplant recipients. We loaded Tac into mesoporous silica nanoparticles (MSN) after which we encapsulated them in the biocompatible, pH-responsive polymer spermine-modified acetalated-dextran (SpAcDEX) [25, 26] through microfluidic nanoprecipitation. The anti-CD4 antibody (CD4Ab) was subsequently attached to the NH_2_ moiety of SpAcDEX to obtain MSN@SpAcDEX-CD4Ab (NP-CD4Ab). With this nanocomposite, Tac was delivered into Tfh cells in a targeted fashion to inhibit their functionality, thereby terminating the activation of B cells to mature into plasma cells. Thus, DSA secretion was reduced, while ABMR injury was suppressed (Figure S2A).

## 2. Results

### 2.1. Construction, Characterization, and Biosafety Analysis of the Nanocomposite

We aimed at constructing nanoparticle systems with immunomodulatory abilities. This study was inspired by our previous study using nanocomplexes formed by complement-targeting antibodies, thermosensitive polymers, and porous nanomaterials [27]. We hypothesized that we could build upgrade nanocomplexes with CD4^+^ cell targeting abilities using pH-responsive polymers and porous nanomaterials. Mesoporous silica nanoparticles (MSN) exhibit controllable pore sizes, high specific surface areas, large pore volumes, and well-established surface modification chemistry, which is a highly desirable characteristic for efficacious drug delivery systems [28–30]. Since 2001, mesoporous silica has been accepted as highly attractive drug carriers. As revealed by transmission electron microscopy (TEM), the prepared MSN [31] had an average particle size of around 80 nm (Figure S2B) with a zeta potential of −15.1 ± 0.5 mV at neutral pH aqueous solution. To avoid premature release of the loaded cargo from MSN, SpAcDEX is used for sealing the pores [32]. SpAcDEX is derived from dextran, a homopolysaccharide of glucose that appears to be well suited for use as a polymeric carrier due to its biodegradability, wide availability, and ease of modification. SpAcDEX, which was prepared as shown in Figure S2C, was deposited onto the nanoparticles by varying its solubility in miscible solutions with the help of microfluidic techniques which is promising for both nano- and microfabrication and encapsulation because of its advanced properties [20, 25, 33]. The two phases' solutions were mixed in a microfluidic chip to obtain a Tac@MSN@SpAcDEX (Tac@NP) precipitate (Figure S2D), and the core/shell structure between MSN and SpAcDEX was observed by TEM (Figure S2E). Tac was loaded in MSN in ethanol at a loading capacity of up to 438.2 mg/g (Figure S3A). The *in vitro* release profile of the Tac encapsulated in nanocomposites in PBS buffer is as shown in Figure S3B, with concentrations calculated from standard curves against different pH values. Under physiological conditions, the drug contained in nanocomposites exhibited a 2.5% cumulative release (2.20 ± 0.29%) within 24 h. Due to the stability exhibited by SpAcDEX at pH 7.4, there were no significant changes in release over the subsequent time period. In contrast, the rapid release of Tac in an acidic environment (pH = 5.0) corresponded to degradation of the SpAcDEX polymer on the surface of nanocomposites. At pH 5, the nanocomposite exhibited an efficient cumulative release of 70.32 ± 14.32% over 48 h. Thus, the release of Tac is triggered by intracellular acidic environments, such as those in endosomes and lysosomes, which provides a mechanism for controlling the flux of Tac release to achieve the desired biological effects.

To concentrate Tac within the immune germinal center (GC) region to avoid uncontrolled distribution of immunosuppressants, an anti-mouse CD4 monoclonal antibody (CD4Ab) was conjugated on the MSN@SpAcDEX (NP) surface to enhance CD4^+^ cell-targeting abilities. Nanocomplex surface-targeted functionalization was facilitated by anchoring CD4Ab on the outer polymer coating [34]. Bonding efficiency of CD4Ab was quantified by SDS polyacrylamide gel electrophoresis (PAGE) and Coomassie blue dye staining through comparisons with bovine serum albumin standards (Figure S3C). After the reactions, almost all antibodies (>95%) were found to have coupled with NP, and only a small number of antibodies were freely present in the supernatant. Cytotoxicities of Tac, NP, and Tac loaded NP were assessed for viability measurements by the WST-1 assay (Figure S3D). None of the components, i.e., Tac, NP, and Tac@NP, exhibited significant toxicities at 10 to 100 *μ*g/ml. Tac@NP alone exhibited little toxicities to splenocytes from 10 to 100 *μ*g/ml. There was no difference in toxicity between the Tac and nanocomplex carried Tac in the tested concentration range. These findings indicate that MSN can efficiently load Tac and be further encapsulated with biocompatible polymers to form pH-responsive nanocomplexes.

### 2.2. Helper T Cell (CD4^+^) Specific Targeting and Uptake *In Vitro*

Tfh cells play an essential role in antibody-secreting plasma cell generation. Tfh development starts from naïve CD4^+^ cell activation, which is stimulated by dendritic cells presenting MHC peptides [9]. Activated naïve CD4^+^ cells upregulate Bcl-6 and CXCR5 expressions and further differentiates into Tfh cells [7]. In the GC selection process, GC light zone B cells (centrocytes) are selected by survival signals derived from Tfh cells, which mediates the differentiation of centrocytes into plasmablasts or activates apoptosis [35, 36]. The sufficiency of Tfh cell number and activation to MHC peptides enhances plasma cell generation [7]. Thus, in this study, we selectively inhibited CD4^+^ cell activation and its maturation into Tfh to suppress ABMR.

**Figure 1:**
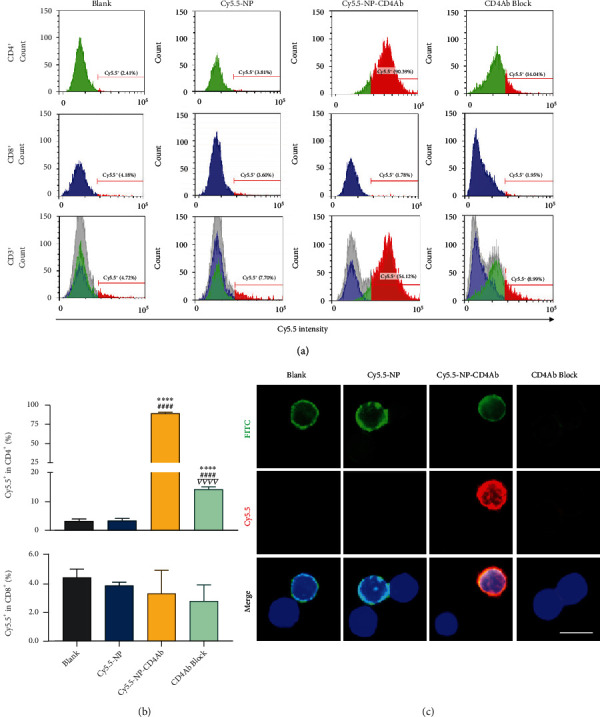
Detection of nanocomposites binding specificity in CD4^+^ T cell. Splenocyte suspensions were incubated with PBS (blank), Cy5.5-NP, and Cy5.5-NP-CD4Ab without (Cy5.5-NP-CD4Ab) or with (CD4Ab block) CD4Ab preincubation. CD4Ab-FITC was added with Cy5.5-NP and Cy5.5-NP-CD4Ab at 1 : 1 ratio simultaneously. (a) Cy5.5 signal intensities in helper T cells (CD4^+^), cytotoxic T cells (CD8^+^), and T cells (CD3^+^) were detected using flow cytometry. (b) Percentages of Cy5.5 positive (Cy5.5^+^) cells in CD4^+^ and cytotoxic T cell (CD8^+^) are shown as mean ± SD. ^∗∗∗∗^*p* < 0.0001 vs. blank group; ^####^*p* < 0.0001 vs. Cy5.5-NP group; ^∇∇∇∇^*p* < 0.0001 vs. Cy5.5-NP-CD4Ab group. (c) Cell surface-conjugated Cy5.5-NP (red) and immunofluorescence staining of CD4Ab-FITC (green) were visualized with fluorescence imaging. Nuclei were stained with Hoechst 33342 (blue). Bar = 10 *μ*m.

An ideal improved drug delivery system should have enhanced targeting effects to the cells of interest; therefore, the binding affinity of particles delivered to CD4^+^ cells was investigated. Red fluorescent dye (sulfo-cyanine 5.5 NHS ester (Cy5.5)) labelled nanoparticles were used for *in vitro* and *in vivo* targeting experiments. The Cy5.5-NP and Cy5.5-NP-CD4Ab binding specificity in primary splenocytes was initially evaluated by flow cytometry, with the gating strategy shown in Figure S4. After incubation with Cy5.5-NP-CD4Ab, the Cy5.5 signals were found to be dominant in the CD4^+^ cell population but were markedly attenuated by preincubation with high concentrations of the CD4 antibody (Figures 1(a) and 1(b)). In cytotoxic T (CD8^+^) cells, Cy5.5 signals did not exhibit any significant differences among all groups and maintained at a low level. Immunofluorescence images confirmed that Cy5.5 signals in the Cy5.5-NP-CD4Ab group were colocalized with CD4Ab-FITC on the cell surface; however, after CD4 antibody preincubation, both Cy5.5 and FITC signals were markedly inhibited (Figure 1(c)). In the blank group that had not been treated with Cy5.5-NP, a small population of Cy5.5 positive cells was found, which might have been due to noise signals of the flow cytometer. The CD3^+^ population merged the signals from CD4^+^ and CD8^+^ cells, and the result also confirmed the existence of the double negative (CD4^−^/CD8^−^) population. Aggressive competitive inhibition of homologous antigens demonstrated that the affinity of Cy5.5-NP-CD4Ab was mainly depended on well-specific antigen targeting and ensured the preservation of CD8^+^ cell activities.

### 2.3. CD4^+^ Cell-Specific Targeting *In Vivo*

**Figure 2:**
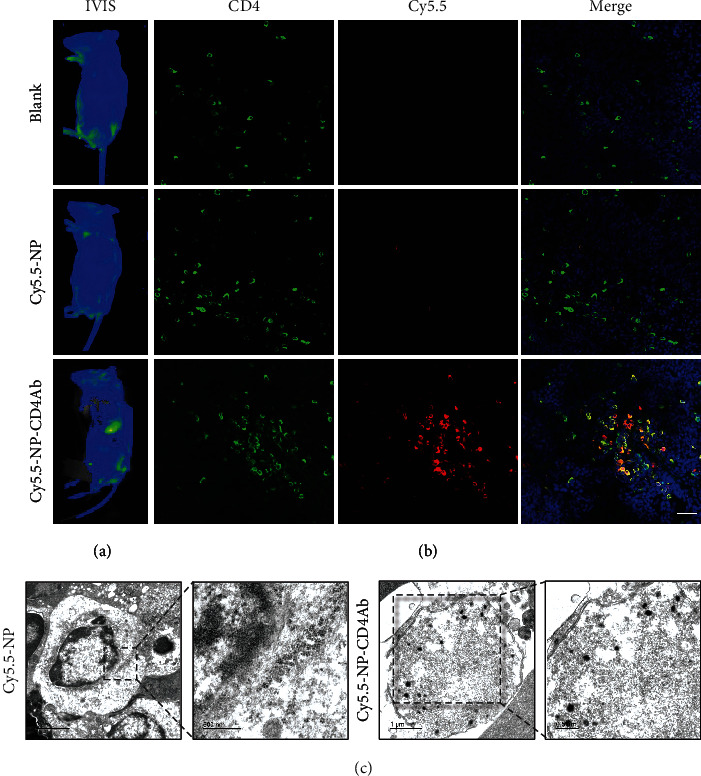
Nanocomposites targeting efficiency evaluated in vivo. (a) In vivo fluorescence imaging of mice using IVIS spectrum system. Saline, Cy5.5-NP, or Cy5.5-NP-CD4Ab was injected via tail vein into C57BL/6 mice. Animals were illuminated with light using Cy5.5 channel (bright green signal). (b) Frozen sections from the spleen were stained with anti-mouse CD4-FITC (green), NP were labeled with Cy5.5 (red), and nuclei were stained with Hoechst 33342 (blue). Bar = 25 *μ*m. (c) TEM images of spleens from mice injected with Cy5.5-NP or Cy5.5-NP-CD4Ab.

*In vivo* NP-CD4Ab affinity was further analyzed in C57BL/6 mice. At 4 h after tail vein injection of saline, Cy5.5-NP, and Cy5.5-NP-CD4Ab, the Cy5.5 signals were monitored by an *in vivo* imaging system (IVIS). Signals were strongly emitted from the spleens of Cy5.5-NP-CD4Ab-injected mice, while no detectable signals were observed in the spleens of saline and Cy5.5-NP-injected groups (Figure 2(a)). Through CD4Ab-assisted targeting, Cy5.5 signals accumulated in the spleen, confirming the specific tissue targeting ability of Cy5.5-NP-CD4Ab in *in vivo* environments.

Mice spleens were harvested after *in vivo* imaging with IVIS, after which immunofluorescence was used to investigate cellular targeting. Abundant Cy5.5-NP-CD4Ab were found to have accumulated in spleen sections colocalized with CD4, while little amounts of Cy5.5-NPs were found in spleen sections at random patterns (Figure 2(b)). Thus, specific *in vivo* targeting of Cy5.5-NP-CD4Ab depended on the affinity of the ligated antibody, while signal density is affected by positive correlations of the abundance of the target. CD4^+^ cells are distributed in the bone marrow and lymphoid tissues and are especially enriched in the spleen, as confirmed by Cy5.5 signal distribution. Targeting specificity of Cy5.5-NP-CD4Ab was confirmed by TEM. Cy5.5-NP-CD4Ab were clearly observed in the cytoplasm of splenocytes, but no Cy5.5-NP particles were found, indicating the importance of CD4Ab functionalization for splenocyte endocytosis via a target-mediated process (Figure 2(c)). Therefore, *in vivo* specific targeting of NP-CD4Ab was successful. During ABMR pathogenesis, the spleen is the major region of B cell maturation, and cell proliferation can easily be detected in the spleen, even only by gross pathological phenotype. In this regard, the bone marrow and lymph nodes were not included in this study.

### 2.4. Donor-Specific Antibody and C4d Deposition Assay

**Figure 3:**
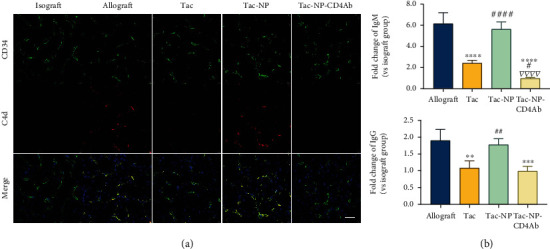
C4d deposition detection and serologic donor-specific antibody assay. C57BL/6 mice recipients received C57BL/6 donor kidney (isograft) and Balb/c donor kidney (allograft). Allograft recipients were administrated with saline (allograft), free Tac, nanocomplex capsuled Tac (Tac-NP), or CD4Ab binding NP-Tac (Tac-NP-CD4Ab) daily. The dosage of Tac was equivalent to 1 mg/kg. (a) Frozen sections of grafts were immunofluorescently stained with CD34 (green) and C4d (red) antibody. Bar =25 *μ*m. (b) Levels of immunoglobulin G (IgG) and immunoglobulin M (IgM) isotypes in donor-specific antibody (DSA) at the end point of the experiment were measured with flow cytometry and mean fluorescence intensities of IgM and IgG were represented as fold change values in isograft group (mean ± SD). ^∗∗^*p* < 0.01, ^∗∗∗^*p* < 0.001, and ^∗∗∗∗^*p* < 0.0001 vs. the allograft group; ^#^*p* < 0.05 and ^##^*p* < 0.01 vs. the Tac group; ^∇∇∇^*p* < 0.001 vs. the Tac-NP group.

In the ABMR process, alloantigens on the endothelium surface are recognized and bind circulating donor-specific antibodies (DSA). By binding complement subcomponent C1q, Fc fragments of DSA complement cascade activation induce multiple inflammatory and rejection cascades [37]. As a split product of the complement, capillary C4d deposition in transplanted organs is a stronger proof of humoral rejection, relative to circulating DSA, and is the most important pathological marker for ABMR diagnosis. In a recent study, an immunofluorescence assay was used to visualize C4d in allografts. After presensitization with alloskin transplantation, transplanted allokidney from the same donor resulted in acute ABMR attack [38]. After 5 days of transplantation, we found marked linear circumferential C4d depositions on peritubular capillaries (CD34^+^ cells) in transplanted allografts (Figure 3(a)), consistent with findings from previous investigations on ABMR [39]. Weak C4d signals were found in isografts, and they correlate with complement activation and C5b-9 formation by ischemia/reperfusion injury during surgery [40]. As the first choice of immunosuppressive regimen against rejection, Tac suppressed the C4d deposition significantly in allograft endothelium. Delivery of Tac with NP-CD4Ab (Tac-NP-CD4Ab) significantly inhibited C4d deposition to nearly invisible. In Tac-NP group, the allograft exhibited moderate C4d deposition, probably due to slowdown and nontargeting drug release from Tac-NP nanocomposites.

Serologic evidence of circulating DSA is a diagnostic index for active ABMR [41–43]. Immunoglobulin M (IgM) and immunoglobulin G (IgG) antibodies are two major DSA subclasses, and the IgM isotype is associated with refractory ABMR [44]. In early occurrent ABMR, IgM DSA is the pioneer immunoglobulin subset for humoral immune responses, which undergoes IgG isotype switch in GC at the meantime [45]. In this study, IgM and IgG titers were evaluated Figure 3(b). At 2 weeks after skin transplantation, a gradual decline in IgM and IgG levels has been reported [46]. The differences in IgM and IgG levels among the groups before kidney transplantation were not marked (data not shown) in this study. Abundant IgM and IgG levels were noted in kidney recipients, reaching around 6 times and 1.5 times, respectively, relative to isograft recipients. This shows that isotype IgM DSA is potentially the immunoglobulin class switch source of isotype IgG after transplantation in acute phase ABMR. Daily Tac administration significantly suppresses IgM and IgG titers. Tac-NP has weak effects on both IgM and IgG reduction, relative to allograft group. Tac-NP-CD4Ab had IgG secretion inhibition abilities comparable to those of free Tac, with an even stronger inhibition of IgM than in Tac group, which was also consist with the finding that IgM-secreting memory B cells are more sensitive to immunosuppressant than IgG-secreting B cells [47]. CD4^+^ cells in GC are responsible in controlling immunoglobulin class switching, somatic hypermutation of immunoglobulin variable region genes, and secretion of high affinity antibodies [48]. The reduction of IgM and IgG levels reflects impaired affinity maturation and class switch due to the detrimental effect of Tac-NP-CD4Ab on CD4^+^ cells. These events mainly occur in GC in secondary lymphoid tissues. Thus, targeted delivery of Tac can potentially reduce the refractory period of ABMR.

### 2.5. Tfh Differentiation and PC Generation

Tfh cells differentiate from naïve CD4^+^ cells by defined expression elevations of B cell lymphoma 6 (Bcl-6) and other cell surface markers, such as CXCR5 and PD1. Tac inhibits T cell development by impairing nuclear factor of activated T cell signals, which play crucial roles in T cell development and function. To assess the suppression effects of delivered Tac, CXCR5^+^Bcl-6^+^ Tfh cell population in CD4^+^ cells from the spleen were quantified by flow cytometry (Figure S5).

Under ABMR pathological conditions, maturation and proliferation of B cells into effective B cells, such as PC as well as memory B cells, and antigen class switching in effective B cells are Tfh cell-dependent processes in GC [49–51]. Therefore, PC proportions were evaluated to establish the GC selection process.

**Figure 4:**
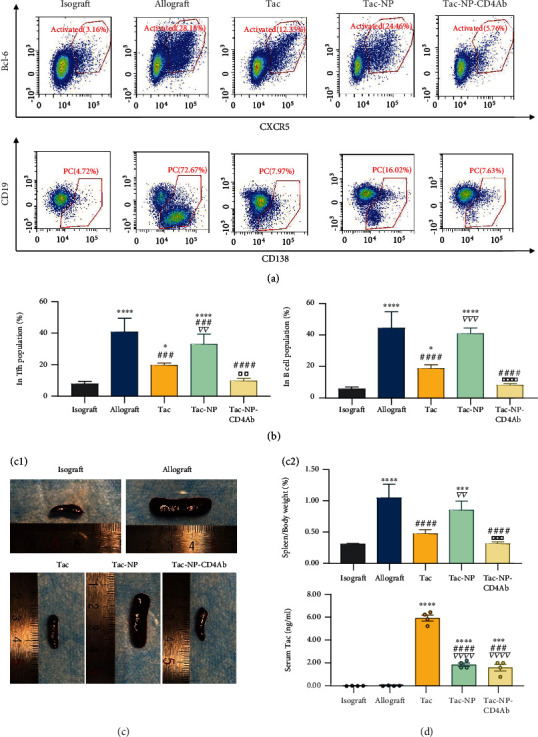
The impact on follicular helper cells (Tfh) and plasma cells (PC). (a) Flow cytometry analysis of Tfh activation and plasma cell in the spleen after transplantation. Three days after renal transplantation into C57BL/6 mice, allograft recipients were treated with PBS (allograft), Tac, nanocomplex capsuled Tac (NP-Tac), and CD4Ab binding NP-Tac (Tac-NP-CD4Ab) daily. Bcl-6^+^ CXCR5^+^ CD4 T cell and CD19^low/int^ CD138^+^ B220 B cell were sorted. (b) Quantitative proportion analysis of Bcl-6^+^ CXCR5^+^ population in CD4^+^ T cell and CD19^low/int^ CD138^+^ population in B220^+^ B cells was calculated and shown as mean ± SD. (c) The growth of the germinal center after transplantation was reflected by volume and weight of the recipient's spleen. Spleens were harvested from recipients and intact mice bodies and weighted. Spleen weight/body weight percentage was shown as mean ± SD. (d) Tac concentration in serum was measured after 2 h of injection. ^∗^*p* < 0.05, ^∗∗^*p* < 0.01, ^∗∗∗^*p* < 0.001, and ^∗∗∗∗^*p* < 0.0001 vs. the isograft group; ^###^*p* < 0.001 and ^####^*p* < 0.0001 vs. the allograft group; ^∇∇^*p* < 0.01 and ^∇∇∇∇^*p* < 0.0001 vs. the Tac group; ^□□^*p* < 0.01, ^□□□^*p* < 0.001, and ^□□□□^*p* < 0.0001 vs. the Tac-Np group.

After allograft transplantation, Tfh levels in CD4^+^ cells were markedly elevated (*p* < 0.0001 vs. isograft group), accompanied by increased PC levels in B cells (*p* < 0.0001 vs. isograft group), consistent with a sharp increase in DSA (Figures 4(a) and 4(b)). Tac administration reduced the levels of activated Tfh and PC populations, compared to the allograft group. Tac-NP partially eliminated the suppression effects of Tac, with slight elevations in Tfh and PC proportions, relative to Tac group. Besides, CD4Ab-coated NP (Tac-NP-CD4Ab) significantly enhanced the inhibitory effects of Tac on GC selection process, showing lowest proportions of Tfh and PC among allograft KT groups. Tac exerted weaker effects on Tfh cells than cyclosporine on *de novo* DSA [52, 53]; however, our findings show sufficient therapeutic benefits of targeted delivery of Tac by NP-CD4Ab.

In GC selection, Tfh cells are stimulated by mature B cells to form a positive feedback loop in cell number growth and survival signal strength. Thus, GC size correlates with the number of activated Tfh cells [12]. Spleen mass in the allograft group increased by more than three times, compared to the isograft group. Interestingly, Tac or Tac-NP-CD4Ab effectively depleted spleen mass growth under ABMR (*p* < 0.0001 vs. allograft group). In the absence of CD4Ab targeting, Tac-NP showed a weak effect (*p* < 0.01 vs. Tac group and *p* < 0.001 vs. Tac-NP-CD4Ab group, Figure 4(c)). By quantifying serum Tac concentrations (2 h postinjection), lower drug concentrations were noted in Tac-NP and TAC-NP-CD4Ab groups, since NP is stable at serum pH (Figure 4(d)). Thus, premature Tac release was inhibited by NP in serum, while stimulated release happened at target sites after NP endocytosis by target cells. This might lead to weakened suppressive effects of nontargeted Tac-NP, compared to Tac.

### 2.6. Histological Manifestation and Renal Function Assessment

Histopathological manifestations in grafts on day five posttransplant were characterized by two experienced pathologists, according to Banff 2019 [54]. Mild inflammatory infiltration was observed in transplant kidneys of the isograft groups.

**Figure 5:**
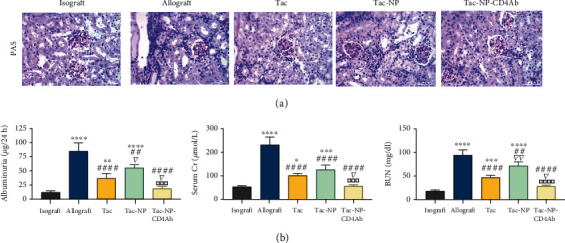
Graft histology and renal function. (a) PAS staining of graft tissues from isograft recipients or allograft recipients with PBS (allograft), Tac, nanocomplex capsuled Tac (Tac-NP), and CD4Ab binding NP-Tac (Tac-NP-CD4Ab). Bar = 25 *μ*m. (b) Protein (albuminuria), serum creatinine (Cr), and blood urea nitrogen (BUN) extracted from 24 h urine were measured for renal graft function. All data are shown as mean ± SD. ^∗^*p* < 0.05, ^∗∗^*p* < 0.01, ^∗∗∗^*p* < 0.001, and ^∗∗∗∗^*p* < 0.0001 vs. isograft group; ^##^*p* < 0.01 and ^####^*p* < 0.0001 vs. allograft group; ^▽^*p* < 0.05 and ^▽▽^*p* < 0.01 vs. Tac group; ^□□□^*p* < 0.001 and ^□□□□^*p* < 0.0001 vs. Tac-Np group.

In the allograft group, cast and cellular tubular and interstitial infiltrates were apparent in the graft, and glomeruli as well as peritubular capillaries were dilated with marginated mononuclear cells and swollen endothelial cells (Figure 5(a)). Consistent with histopathologic manifestations, significant elevations of urinary protein excretion (albuminuria), serum creatinine (Cr), and blood urea nitrogen (BUN) were observed in the allograft group (*p* < 0.0001 vs. isograft group, Figure 5(b)), indicating declined renal functions after acute rejection. There was partial remission of microvascular inflammation in the allograft after administration of Tac or Tac-NP, despite the presence of tubulitis and interstitial inflammation. Improved allograft functions (albuminuria, serum Cr, and BUN) were noted in Tac and Tac-NP groups, relative to the allograft group. Great remission of cellular infiltrations in the allograft was noted in grafts of the Tac-NP-CD4Ab group, accompanied by striking preservation of renal functions (*p* > 0.05 vs. isograft group), implying that Tac-NP-CD4Ab can effectively diminish ABMR induced allograft histological and functional injury.

### 2.7. Side Effects of Nanocomposites

Systemic effects of Tac with or without NPs were evaluated by assessing body weight changes and H&E staining of organs from each group. Differences in body weights among the control, Tac, Tac-NP, and Tac-NP-CD4Ab groups were insignificant (Figure S6). Histologically, there were no lesions, edema, or inflammation in the lungs, liver, spleens, kidneys, and brains of mice (Figure S7A).

**Figure 6:**
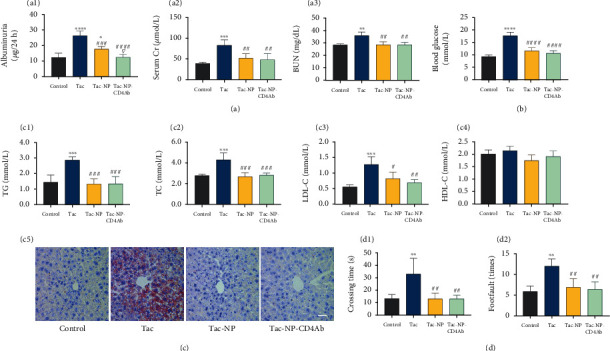
Side effect evaluation. Side effects of 4-week administration of saline, Tac, Tac@MSN@SpAcDEX (Tac-NP), and Tac-NP-CD4Ab were evaluated in healthy adult C57BL/6 mice (*n* = 5 for each group). (a1–a3) Protein (albuminuria), serum creatinine (Cr), and blood urea nitrogen (BUN) extracted from 24 h urine were measured for nephrotoxicity evaluation. (b) Blood glucose concentration was determined with an automatic blood glucose meter for the determination of posttransplant diabetes mellitus. (c1–c5) Triglycerides (TG), total cholesterol (TC), low-density lipoprotein cholesterol (LDL-C), and high-density lipoprotein cholesterol (HDL-C) in serum were measured for lipid metabolic dysfunction detection. Frozen liver sections were stained with Oil Red O for lipid droplets accumulation (bar = 50 *μ*m). (d1 and d2) Motor coordination performance of mice was assayed using beam walking test. The average crossing time and the number of foot slips on the beam (footfault) were recorded. Data are shown as mean ± SD. ^∗^*p* < 0.05, ^∗∗^*p* < 0.01, ^∗∗∗^*p* < 0.001, and ^∗∗∗∗^*p* < 0.0001 vs. control group; ^#^*p* < 0.05, ^##^*p* < 0.01, ^###^*p* < 0.001, and ^####^*p* < 0.0001 vs. Tac group; ^∇^*p* < 0.05 vs. Tac-Np group.

The most common side effect of Tac is nephrotoxicity [55, 56], especially during kidney transplantation. In this study, after 4 weeks, antibody-ligated (Tac-NP-CD4Ab) or antibody free (Tac-NP) nanocomposites carrying Tac exhibited limited nephrotoxicity (Figure 6(a)), low levels of urinary proteins (albuminuria), serum creatinine (Cr), and blood urea nitrogen (BUN), compared to Tac-treated group. Kidney histological changes reflected reduced nephrotoxic injury by nanocomposites. Moderate interstitial fibrosis was noted in Tac group kidneys. In the control, Tac-NP, and Tac-NP-CD4Ab groups, there were no noticeable changes in the kidneys (Figure S7B).

Calcineurin inhibitors (CNI), including Tac and cyclosporine A, are associated with glucose and lipid metabolism dysregulation [57]. Tac-related insulin resistance and hyperglycemia correlated with poor prognosis for solid organ recipients [58, 59]. Posttransplant diabetes mellitus (PTDM) increases cardiovascular risk [60]. In a recent study, after 4-week Tac administration, fasting blood glucose levels of the Tac group increased to 17.90 ± 1.38 mmol/l, which is greater than diabetes diagnosis standards (above 13.3 to 16.7 mmol/l) for mice. The C57BL/6 mice showed a higher susceptibility to glycemic dysregulation of Tac and more stress than other mice strains [61], while the increase in fasting blood glucose levels for Tac-NP and Tac-NP-CD4Ab groups was slight, compared to saline treated (control) group (Figure 6(b)). Hyperlipidemia is also a side effect of Tac; thus, triglycerides (TG), total cholesterol (TC), low-density lipoprotein cholesterol (LDL-C), and high-density lipoprotein cholesterol (HDL-C) levels were measured at the end point of the toxicity assay. Mice in nanocomposite treated groups, both Tac-NP and Tac-NP-CD4Ab groups, had low concentrations of TG, TC, and LDL-C, relative to those in the Tac group (Figures 6(c1)–6(c4)). Oil Red O staining showed that lipid deposition (red droplets) in the Tac group was significantly higher than in Tac-NP and Tac-NP-CD4Ab groups, and Tac-NP group showed sporadic distributions of fat deposition, while the control and Tac-NP-CD4Ab groups showed undetectable lipid droplets (Figure 6(c5)). Thus, Tac-NP-CD4Ab prevented uncontrollable increase in glycemia and lipid metabolism dysregulation.

Compared to non-CNI, such as sirolimus and other CNI, such as cyclosporine A, Tac induced more frequent tremors (82%) in kidney transplantation patients [62, 63]. Most of the Tac inducing tremors are action tremors, and their severity is associated with peak serum concentrations of Tac. A previous pharmacokinetic study in mice showed that Tac reaches peak whole blood drug concentrations at around 2 h postadministration [64]. Thus, the beam walking test was used to measure fine motor functions of mice after 2 h of drug injection. Mice in the Tac-NP and Tac-NP-CD4Ab groups spend less crossing time and had fewer footfaults through the beam, than mice in the Tac group (Figure 6(d)), suggesting that mice administered with Tac-NP or Tac-NP-CD4Ab had less tremors and might be associated with decreased Tac fluctuations in serum and preserved motor neuron function.

In clinical practice, serum Tac concentrations should be closely monitored for their therapeutic significance. High concentrations are associated with nephrotoxicity, neurotoxicity, metabolic disorders, and nonspecific immunosuppression. Low concentrations lead to rejection and allograft loss. In this study, Tac-NP-CD4Ab doses were based on Tac mass for effective comparisons; however, for targeted delivery, an excess dose was also used for ABMR suppression. We found reduced side effects in the long-term toxicity study. Targeting of Tfh cells by immunosuppressant nanoparticles during mice kidney transplantation relieved antibody-mediated alloreactivity while significantly limiting the adverse effects of Tac.

## 3. Conclusion

Overall, the proposed nanotechnology can overcome the challenges associated with ABMR after organ transplantation by enhancing the local potency of immunosuppressive cargos aimed at CD4^+^ cells, achieving ABMR suppression and improving long-term allograft survival. These outcomes are due to various factors, including nanoparticle encapsulation to protect the cargo from degradation before reaching its target cells, directing cargo to cells of interest, and promoting cargo accumulation in specific subcellular compartments. Our results show that the dose of immunosuppressant in nanoparticles targeting CD4^+^ cells which significantly inhibited plasma cell generation reduced DSA secretion, and allograft injury was much lower than those administrations inducing side effects without encapsulation and controls cargo release. This effect allowed us to overcome many of the delivery challenges encountered in previous studies and with the bonus of low incidences of side effects after long-term medical administration. Based on the strategy in our study and with high-resolution antigenic epitope mapping of Tfh cells at the germinal center, ABMR therapy will be improved.

## 4. Materials and Methods

### 4.1. Materials

Cetyltrimethylammonium chloride solution (CTAC, 25 wt%), tetraethyl orthosilicate (TEOS), triethanolamine (TEA), (3-aminopropyl)triethoxysilane (APTEOS), tacrolimus (Tac), ammonium nitrate (NH_4_NO_3_), dextran (Mw 9-11 000 g/mol, from Leuconostoc mesenteroides), 2-methoxypropene, and spermine were purchased from Sigma-Aldrich, while sulfo-Cy5.5 was purchased from Lumiprobe Corporation. All chemicals were used as received without further purification.

### 4.2. Animals

Animal experiment protocols were approved by the Research Ethics Committee of the First Affiliated Hospital, College of Medicine, Zhejiang University (No. 2019-1231) and were performed according to the National Institutes of Health Guide for the Care and Use of Laboratory Animals (NIH Publication No. 80-23). Eight-week-old male BALB/c and C57BL/6 mice (weight: 23 ± 2 g) were purchased from the Laboratory Animal Center of Zhejiang University. They were maintained in a controlled environment (25°C), a 12 h light/dark cycle, and were allowed unrestricted access to water and standard rodent chow.

### 4.3. Synthesis of Mesoporous Silica Nanospheres (MSN)

First, 24 ml CTAC (25 wt%) solution and 0.18 g TEA were added to 36 ml water under slow stirring at 60°C for 1 h. Then, 20 ml (10 *v*/*v*%) TEOS cyclohexene solution was carefully added to the above mixture of water, CTAC, and TEA. The reaction was conducted overnight at 60°C in an oil bath with magnetic stirring at 150 rpm to obtain the product. The product was collected by centrifugation and washed several times using ethanol to remove residual reactants. Then, the collected product was dispersed in 0.6 wt% NH_4_NO_3_ ethanol solution and extracted twice at 60.0°C for 6 h each to remove the template. The resulting MSN was kept in ethanol and stored at 4°C for subsequent experiments. Sulfo-Cy5.5-NHS (Cy5.5) labelled MSN (MSN-Cy5.5) nanostructures were synthesized using a similar process as MSN, but the CTAC solution contained additional Cy5.5 (1.0 mg/ml, 10 *μ*l).

### 4.4. Synthesis of Spermine-Modified Oxidized Acetalated Dextran (SpAcDEX)

1.0 g of dextran with an average molecular weight of 9000-11000 was dissolved in 4 ml water, after which 0.22 g sodium periodate was added to the above solution and stirred for 5 h at room temperature to produce partially oxidized dextran. Partially oxidized dextran was further purified by multiple dialysis with distilled water through a regenerated cellulose membrane (MW 3500 Da). After lyophilization, a white partially oxidized dextran powder was obtained. 1.0 g of the partially oxidized dextran was added to 10 ml anhydrous dimethyl sulfoxide (DMSO) and stirred until complete dissolution, followed by the addition of pyridinium p-toluenesulfonate (15.6 mg, 0.062 mmol) and 2-methoxypropene (3.4 ml, 37 mmol), which had been sealed under N_2_ protection to prevent its evaporation. After 3 h, the reaction was terminated by adding 1 ml TEA after which the modified partially oxidized dextran was precipitated by adding 100 ml distilled water. The product was separated by centrifugation at 10000 rpm for 10 min and the resulting precipitate washed using deionized water by sonication dispersion, followed by centrifugation and removal of the supernatant. A white powder of partially oxidized acetalated dextran was obtained by lyophilisation. Then, 1.0 g of partially oxidized AcDEX was dissolved in 10 ml anhydrous DMSO, mixed with 4.0 g of spermine, and reacted at 50°C for 24 h. NaBH_4_ (2.0 g) was added to the mixture and the reduction performed for a further 18 h at room temperature. SpAcDEX was precipitated by adding 40 ml of deionized water to the above solution and separated by centrifugation at 10000 rpm for 10 min. The resulting precipitate was washed several times using deionized water. After lyophilisation, SpAcDEX was obtained and stored at -20°C.

### 4.5. Fabrication of a Three-Dimensional Microfluidic Coflow Device

The three-dimensional microfluidic coflow device was fabricated as previously described. Borosilicate glass capillaries of different parameters were tailored and assembled on glass slides. One end of the cylindrical capillary (inner and outer diameters of 580 and 1000 *μ*m, respectively, World Precision Instruments, Inc., United States) that was used as the inner tube was first tapered (20 *μ*m diameter) using a micropipette puller (P-97, Sutter Instrument Co., United States); then, the orifice diameter was enlarged to 80 *μ*m using a sandpaper. The manufactured inner tube was inserted into another thicker cylindrical capillary outer tube (inner and outer diameters of 1100 and 1500 *μ*m, respectively, Vitrocom, Inc., United States) and assembled in a coaxial configuration. The outer capillary was terminated by two hypodermic needles (Warner Instruments, United States). Where necessary, a transparent epoxy resin (5 min Epoxy, Devcon) was used to seal the assembly. The two miscible liquids were separately injected into the microfluidic device at a constant flow rate through a polyethylene tube connected to syringes. Flow rates of the different liquids were controlled by pumps (PHD 2000, Harvard Apparatus, United States).

### 4.6. Fabrication and Characterization of the SpAcDEX-Encapsulated MSN

To encapsulate MSN into the SpAcDEX polymer, 1.0 mg/ml MSN and 5 mg/ml SpAcDEX in ethanol were mixed as the internal phase of the microfluid, with 1% PVA aqueous solution being used as the outer phase. The two-phase solutions were injected into the above fabricated microfluidic device through polyethylene tubes connected to syringes at a flow rate of 2 ml/h for the inner phase and 40 ml/h for the outer phase. The flow rate was controlled by a microfluidic pump. The MSN@SpAcDEX (NP) nanocomposites precipitate obtained after mixing the two-phase solutions was collected in 0.1% PVA solution with gentle agitation at 200 rpm. Then, centrifugation was performed at 10000 rpm for 5 min, followed by washing several times using deionized water. NP were stored in deionized water at 4°C. Structures of synthesized MSN and NP were evaluated by a transmission electron microscope (TEM, JEOL 1400 Plus, United States) at an acceleration voltage of 80 kV. The surface zeta potential of NP was measured by a Zetasizer Nano ZS using disposable folded capillary cells (DTS1070, Malvern, UK).

### 4.7. *In Vitro* Tac Loading and Release Assay

For encapsulation of Tac, it was loaded into MSN nanoparticles using the immersion method. First, a 5.0 mg/ml ethanol solution of Tac was prepared, followed by resuspension of 1.0 mg MSN in this solution, resulting in a mass ratio of 1 : 5 (MSN/Tac). The mixture was slowly stirred for 24 h at room temperature to reach saturation of MSN loading. Then, the precipitate was acquired by centrifugation, washed 3 times using ethanol to obtain the MSN loaded with Tac (Tac@MSN). All Tac in the supernatant was carefully collected and quantified according to the standard curve of Tac in the ethanol solution. The loading capacity was calculated as follows: Loading capacity% = amount of Tac in MSN/MSN × 100%.

*In vitro* release of Tac from nanocomposites was evaluated in phosphate-buffered saline solutions. pH 7.4 and 5.0 were used to simulate extracellular and intracellular environments, respectively. The specific release profiles were conducted by placing drug-loaded nanocomposites (500 *μ*g) into their respective buffer solutions and shaking at 100 rpm and 37 ± 1°C. Free Tac was used as the control. Due to low solubility of Tac, amphiphilic F-127 (5%, *w*/*v*) was added to all release buffers. Samples were first centrifuged (10,000 rpm, 5 min) at various time points to withdraw 100 *μ*l of the supernatant solution after which the same volume of prewarmed medium was added to replace the withdrawn volume, followed by quantification of drug concentration in the supernatant by HPLC.

### 4.8. Conjugation of Nanocomposites with CD4Ab

To conjugate nanocomposites with CD4Ab, 5 mg nanocomposites (based on SpAcDEX mass) was added to 2 ml freshly prepared 1 mM bis-sulfosuccinimidyl suberate (BS3, Sigma-Aldrich) in PBS reagent (1 mM, pH 7.4). The mixture was continuously stirred with a magnetic bead (200 Hz) for 6 h at room temperature. After washing with PBS twice, BS^3^-conjugated nanocomposites (5 mg/ml in PBS) were incubated with anti-mouse CD4 antibody (0.15 mg/ml, Clone 145-2C11; BD Pharmingen, CA, United States) and stirred overnight at 4°C. Free BS^3^ bases were blocked by 50 mM glycine and stirred for 2 h at room temperature. CD4Ab-conjugated nanocomposites (NP-CD4Ab, Cy5.5-NP-CD4Ab, or Tac-NP-CD4Ab) were precipitated at 10,000 g centrifugation for 5 min, washed with PBS thrice, and diluted to 100 *μ*g/ml with PBS.

### 4.9. *In Vitro* Toxicity Study

Primary splenocytes were used to determine MSN@SpAcDEX nanocomposites toxicity. Water-soluble tetrazolium salt (WST-1) assay was performed following the manufacturer's instructions. Briefly, primary splenocytes (5 × 10^4^ cells/well) were seeded into 96-well microplates and incubated with 0, 10, 15, 20, 30, 50, and 100 *μ*g/ml of Tac (Tac) or Tac-NP containing the same Tac dose in CO_2_ incubator. The NP group contained the same NP mass as Tac-NP group. After incubation for 8 h, WST-1 reagent (10 *μ*l/well, Cat. Cellpro-ro, Roche, Basel, Switzerland) was added and incubated for another 4 h. Then, absorbance was measured at 450 nm and 600 nm using a multimode plate reader (Infinite M1000 Pro, Tecan, NC, United States).

### 4.10. *In Vivo* Toxicity Study

All experimental protocols in this study were approved by the Research Ethics Committee of the First Affiliated Hospital, College of Medicine, Zhejiang University (No. 2019-1231). All animal handling procedures followed the National Institutes of Health Guide for the Care and Use of Laboratory Animals (NIH Publication No. 80-23). Eight-week-old male C57BL/6 mice (weight: 23 ± 2 g) were obtained from the Laboratory Animal Center of Zhejiang University. Mice were housed under a 12 h light/dark cycle in a controlled environment at 25 ± 2°C with free access to water and food. C57BL/6 mice received intravenous injection of Tac, Tac-NP, or Tac-NP-CD4Ab every 2 days for four weeks equivalent to Tac dose of 1 mg/kg. The PBS injection group served as blank control. At the end of four weeks of drug administration, mice underwent the following experiments.

### 4.11. Beam Walking Test

Fine motor coordination in mice was assessed using beam walking test. Briefly, mice were gently placed at the end of a beam lamp (0.5 cm width, 100 cm length, and 50 cm above the desk). We used a cage at the other end of the beam to attract mice. The crossing time and limb slip number were recorded. Each mouse has four rounds of practice before the formal experiment.

### 4.12. Proteinuria Assay

Mice were housed in metabolic cages (Cat.DXL-XS, Gele Laboratory Equipment Co., Ltd., Suzhou, China) for 24 h urine collection. Urine protein levels were quantified by BCA protein assay kit (23227, Peirce, Thermo Fisher, CA, United States) following manufacturer's instruction.

### 4.13. Serum Testing

Serum samples were isolated from whole blood by centrifugation at 3,000 g for 15 min at 4°C. Serum creatinine (Cr), blood urea nitrogen (BUN), triglyceride (TG), cholesterol (TCs), low-density lipoprotein (LDL), and high-density lipoprotein (HDL) were measured using a biochemistry analyzer (DRI-CHEM 7000i, FUJI, Japan) with purpose-made testing slides.

### 4.14. Histopathology

Mice were sacrificed with overdose pentobarbital sodium (250 mg/kg, i.p., Huadong Medicine Co., Ltd., Hangzhou, China) for cardiac perfusion with saline followed by 4% paraformaldehyde. Frozen liver sections were stained with Oil Red O for neutral lipid visualization. Kidney, heart, liver, spleen, lung, and brain paraffin sections were stained with haematoxylin-eosin (HE) staining, with kidney slides stained again with Masson's trichrome (MT) staining. Slide images were captured with micrographic photos (DM4000, Leica, Frankfurt, Germany).

### 4.15. *In Vitro* Targeting Efficacy Study

Primary splenocytes were isolated from C57BL/6 mouse spleen. Briefly, the spleen was pushed through 40 *μ*m cell strainer (Cat. 431750, Corning, NY, United States), centrifuged at 350 g for 5 min, and resuspended in RPMI 1640 medium (Cat. 11835030, Gibco, NY, United States) containing 10% heat inactivated FBS (Cat. 10100139C, Gibco) to 5 × 10^6^ cells/ml. FITC-labeled CD4Ab (FITC-CD4Ab) was processed using FITC conjugation kit (ab102884, Cambridge, UK) following manufacturer's instructions and its concentration adjusted to 100 *μ*g/ml with PBS.

To evaluate the binding specificity of NP-CD4Ab, one-tenth of the volume (i.e., 20 *μ*l) of Cy5.5-NP-CD4Ab and FITC-CD4Ab were added simultaneously into splenocyte suspensions (i.e., 200 *μ*l) previously incubated with or without 10 *μ*g/ml CD4Ab. Splenocytes incubated with one tenth of the volume of Cy5.5-NP and PBS were used as negative and blank control, respectively. After incubation for 1 h at 37°C, cells were washed and resuspended in PBS. NP targeting capability of cells was evaluated using fluorescence image and flow cytometry assays.

### 4.16. Immunofluorescence Staining for *In Vitro* Targeting

Splenocytes were fixed with 4% paraformaldehyde and mounted on adhesion microscope slide (Cat. 188105, Citoglas, China) with Cytospin™ 4 Cytocentrifugation (Thermo Fisher, CA, United States) at 1,000 rpm for 5 min. Nuclei were stained with Hoechst 33342 (Cat. 62249, Thermo Fisher, CA, United States). Fluorescent images were captured using micrographic photos (DM4000, Leica).

### 4.17. Flow Cytometry for *In Vitro* Targeting

Concentration of splenocyte suspension was adjusted to 5 × 10^5^ cells/100 *μ*l with PBS and stained with Fixable Viability Stain (FVS)-440UV (Cat. 566332, BD Biosciences, Oxford, United Kingdom) at 1 : 1000 ratio for 15 min in the dark. Staining was terminated by washing with the same volume of BSA staining buffer (Cat. 554657, BD) twice before surface antigens staining. After preincubation with Fc block (Cat. 553141, BD) at 1 : 50 ratio for 5 min, antibodies from CD45-BV786 (Clone 30-F11, BD), CD3-PE-Cyanine5 (Clone 145-2C11, BD), B220-PE-Cy7 (Clone RA3-6B2, BD), CD4-FITC (Clone RM4-5, BD), and CD8-BV605 (Clone 53-6.7, BD) were added at 1 : 50 ratio and incubated in the dark for 15 min at 4°C. Cells were then washed twice and resuspended in 0.5 ml cold PBS. Cell analysis was performed using a flow cytometry (CytoFLEX LX, Beckman Coulter, CA, United States) and CytExpert analysis software version 2.3 (Beckman Coulter) following manufacturer's instructions.

### 4.18. *In Vivo* Targeting Efficacy Study

Kidney allograft antibody-mediated rejection model was used to evaluate targeting efficacy *in vivo*. C57BL/6 mice were firstly presensitized with Balb/c skin transplantation. Briefly, after being anesthetized with sodium pentobarbital intraperitoneal injection (50 mg/kg, i.p.), C57BL/6 recipient mouse received a 1 cm diameter skin graft on the dorsal area from Balb/c donor mouse. Two weeks after skin transplantation, presensitized C57BL/6 mouse received a kidney graft from Balb/c (allograft) or C57BL/6 (isograft) donor mouse as previously reported [46]. In brief, the left donor kidney was transplanted into the recipient mouse's inferior vena cava, abdominal aorta, and bladder. Cy5.5-NP-CD4Ab or Cy5.5-NP (5 mg/kg, 200 *μ*l) was injected via tail vein before graft reperfusion. Nephrectomy was then performed on the mouse after well graft reperfusion. After 24 h, mice were anesthetized with isoflurane inhalation for *in vivo* spectrum imaging (IVIS) test. Then, mice were sacrificed with overdose pentobarbital sodium (250 mg/kg, i.p.) to harvest the spleen for organ spectrum imaging, immunofluorescence (IF), and transmission electron microscope (TEM) analysis.

### 4.19. *In Vivo* Imaging System (IVIS) Spectrum

Spectrum imaging was performed using IVIS Lumina LT Series III (Perkin Elmer, MA, United States) with a Cy5.5 filter set (excitation 675 nm, emission 720 nm, and exposure time 1 s) to obtain Cy5.5-conjugated NP signals. All images were acquired and analyzed using Living Image version 4.5 software (Perkin Elmer, MA, United States) and displayed in the same scale of fluorescent intensity. Fluorescence emission was normalized to photons per second per centimeter square per steradian (p/s/cm^2^/sr).

### 4.20. Immunofluorescence Staining for *In Vivo* Targeting

Fifteen-micrometer-thick frozen spleen sections were cut using a cryostat (CM1950, Leica, Wetzlar, Germany). The slides were stained with the same staining. The procedure of cell staining was photographed.

### 4.21. Transmission Electron Microscopy (TEM) for In Vivo Targeting

Spleen tissues were fixed with 2.5% glutaraldehyde solution overnight. Spleen tissues were then carefully washed with PBS (pH 7.4) thrice and postfixed with 1% osmium tetroxide for 1 h. Samples were washed with pure water thrice, stained with 2% uranium acetate for 30 min, dehydrated stepwise using 50%-100% ethanol and 100% acetone, permeated in resin and acetone mixture (1 : 1, *v*/*v*) at room temperature for 2 h, and subsequently embedded in resin. Embedded spleen specimens were cut into 70 nm ultrathin sections using Leica UC7 and viewed using a Tecnai G2 Spirit TEM (Thermo Fisher, CA, United States) operating at 80 kV.

### 4.22. Therapeutic Effect Study

Allograft-recipient mice were treated with sterile PBS, Tac (Tac group), Tac@MSN@SpAcDEX (Tac-NP group), or Tac@MSN@SpAcDEX-CD4Ab (Tac-NP-CD4Ab), whereas isograft-recipient mice were treated with sterile PBS injected intravenously daily after transplantation. Tac dosage in the Tac, Tac-NP, and Tac-NP-CD4Ab groups was equivalent to 1 mg/kg. Mice were caged for 24 h urine collection for proteinuria assay at day 4 after transplantation. After final bodyweight was recorded, mice were sacrificed with pentobarbital sodium overdose to harvest blood, spleens, and grafts. Cr and BUN were measured as mentioned before. The spleen and body weight of the mice were measured.

### 4.23. Serum Tac Concentration Measurement

To quantify the concentration of Tac, Emit® 2000 Tac Assay (Cat. 8R019UL, SIEMENS, DE, United States) was used following the manufacturer's instructions.

### 4.24. Immunofluorescence Staining for C4d Deposition Detection

Ten-micrometer-thick frozen graft sections were stained with anti-CD34 (MEC 14.7) (Cat. ab8158, Abcam) and anti-C4d (HP8033, Hycult, Netherland) and labeled with corresponding secondary antibodies.

### 4.25. Donor-Specific Antibody (DSA) Detection

Briefly, splenocytes from Balb/c donor mice were incubated with C57BL/6-recipient mice serum for 30 min at room temperature and then washed with PBS twice. Anti-mouse IgM Alexa Fluor® 647 (Cat. ab150123, Abcam) and anti-mouse IgG DyLight® 488 (Cat. ab96879, Abcam) were used to label DSA binding. Fluorescence intensities were measured using CytoFLEX LX flow cytometry.

### 4.26. Flow Cytometry for Tfh Cell Activation

Activated Tfh cells in the spleen were labeled with FVS-440UV, CD45-BV786, CD3-FITC (Clone 145-2C11, BD), CD4-V500, CD8-BV605, and CXCR5-PE (Clone 2G8, BD) following surface antigen staining procedure. Subsequently, cell pallets were suspended in 1 ml fixation buffer (Cat. 554655, BD) for 15 min under room temperature and washed with 1 ml perm/wash buffer (Cat. 557885, BD) twice. Anti-mouse Bcl-6-Alexa Fluor 647 (Clone K112-91, BD) was added at 1 : 50 ratio and incubated in the dark for 30 min at 4°C. Cells were then washed twice and resuspended in cold PBS for flow cytometry assay. Plasma cells in peripheral blood cells (PBMCs) were labeled with FVS-440UV, CD45-BV786, CD3-FITC, B220-PE-Cy7, CD19-APC-cy7 (Clone 1D3, BD), and CD138-BV421 (Clone 281-2, BD) following the procedure described earlier.

### 4.27. Statistical Analysis

All statistical analyses were performed using GraphPad Prism 9 (GraphPad Inc., CA, United States). Results are displayed as mean ± standard deviation (SD) unless stated otherwise. ANOVA followed by Tukey's posttest was used for multiple group comparisons. *p* < 0.05 was considered statistically significant.

## Data Availability

Data supporting the findings of this study are available in the main text or the supplementary information.

## Supplementary Materials

Supplementary Materials Figure S1: enhanced T follicular helper- (Tfh-) dependent B cell responses in ABMR. Figure S2: the fabrication procedure of nanocomplexes. Figure S3: characterization and biosafety assay of nanocomplexes. Figure S4: gating strategy for nanocomplex targeting detection. Figure S5: gating strategy for Tfh cell activation in spleen and plasma cell population for PBMC detection. Figure S6: body weight gain during 4-week drug administration. Figure S7: histological H&E staining of main organs and Masson's trichrome staining of kidneys.
