# Supplementary material for: Helper T Cell (CD4+) Targeted Tacrolimus Delivery Mediates Precise Suppression of Allogeneic Humoral Immunity
Source: Research (Wash D C). 2022 Jul 16;2022:9794235. doi: 10.34133/2022/9794235 (PMC9343082; doi:10.34133/2022/9794235)
Supplement: Supplementary Materials — Figure S1: enhanced T follicular helper- (Tfh-) dependent B cell responses in ABMR. Figure S2: the fabrication procedure of nanocomplexes. Figure S3: characterization and biosafety assay of nanocomplexes. Figure S4: gating strategy for nanocomplex targeting detection. Figure S5: gating strategy for Tfh cell activation in spleen and plasma cell population for PBMC detection. Figure S6: body weight gain during 4-week drug administration. Figure S7: histological H&E staining of main organs and Masson's trichrome staining of kidneys. [file 9794235.f1.docx]

Title

Helper T Cell (CD4^+^) Targeted Tacrolimus Delivery Mediates Precise Suppression of Allogeneic Humoral Immunity

Targeted Tacrolimus Delivery to Inhibit Allogeneic Rejection

**Authors**

Jia Shen^1,†^, Chang Liu^2,3,†^, Pengpeng Yan^1^, Meifang Wang^1^, Luying Guo^1^, Shuaihui Liu^1^, Jianghua Chen^1^, Jessica M. Rosenholm^2^, Hongfeng Huang^1,^*, Rending Wang^1,4,^*, Hongbo Zhang^2,3,5,^*

**Affiliations**

^1^ Kidney Disease Center, the First Affiliated Hospital, College of Medicine, Zhejiang University; Key Laboratory of Kidney Disease Prevention and Control Technology, Zhejiang Province.

^2^ Pharmaceutical Sciences Laboratory, Faculty of Science and Engineering, Åbo Akademi University, Turku 20520, Finland.

^3^ Turku Bioscience Centre, University of Turku and Åbo Akademi University, Turku 20520, Finland.

^4^ Organ Donation and Coordination Office, the First Affiliated Hospital, School of Medicine, Zhejiang University, Hangzhou 310003, China.

^5^ Department of Orthopaedics, Shanghai Key Laboratory for Prevention and Treatment of Bone and Joint Diseases, Shanghai Institute of Traumatology and Orthopaedics, Ruijin Hospital, Shanghai Jiao Tong University School of Medicine, Shanghai 200025, China.

^†^ These authors contributed equally to this work

Correspondence should be addressed to Hongbo Zhang; [hongbo.zhang@abo.fi](mailto:hongbo.zhang@abo.fi) and Rending Wang; [rd_wangjia@zju.edu.cn](mailto:rd_wangjia@zju.edu.cn)

Supplementary Materials

Figure S1. Enhanced T follicular helper (Tfh)-dependent B cell responses in ABMR.

Figure S2. The fabrication procedure of nanocomplexes.

Figure S3. Characterization and biosafety assay of nanocomplexes.

Figure S4. Gating strategy for nanocomplex targeting detection.

Figure S5. Gating strategy for Tfh cells activation in spleen and plasma cell population for PBMC detection.

Figure S6. Body weight gain during 4-week drug administration.

Figure S7. Histological H&E staining of main organs and Masson’s trichrome staining of Kidneys.


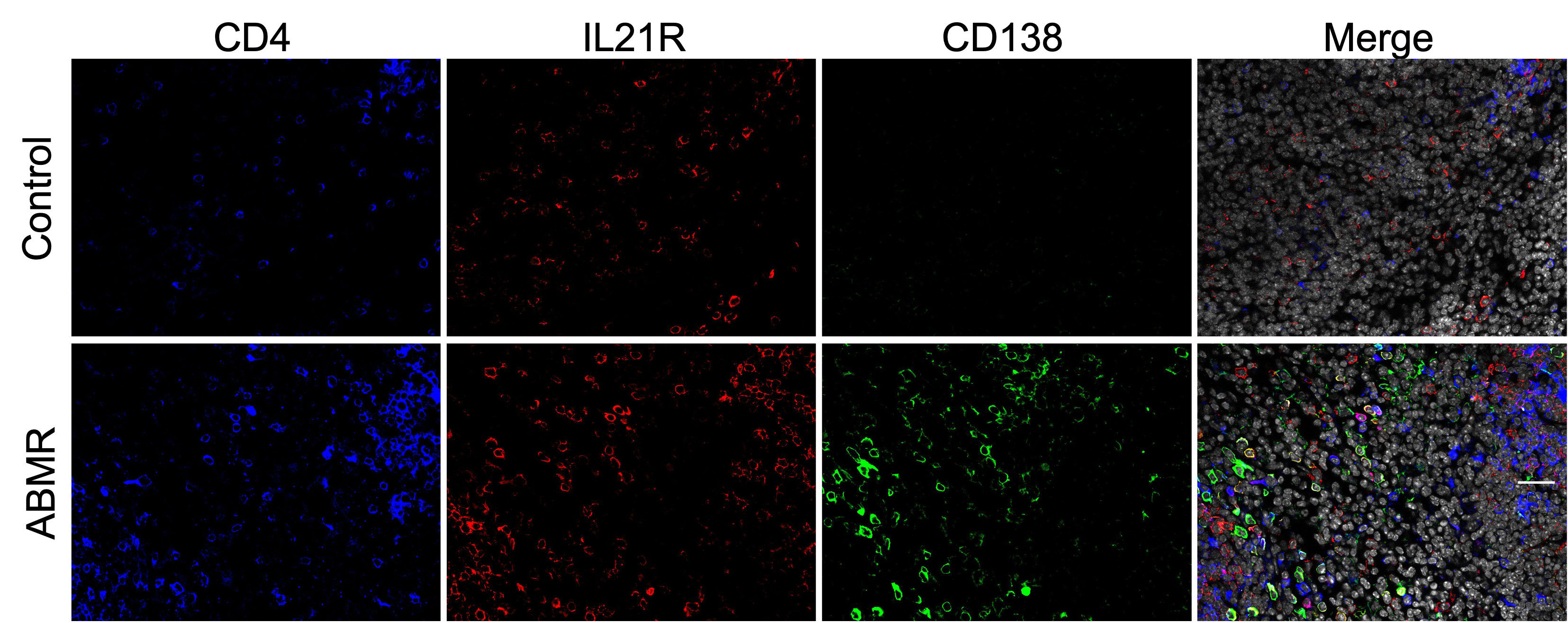


***Figure S1.*** ***Enhanced T follicular helper (Tfh)-dependent B cell responses in antibody-mediated rejection (ABMR).*** *Immunofluorescence staining with anti-mouse CD4 (blue), IL21R (red), CD138 (Green) and DAPI. Bar = 25 μm.*


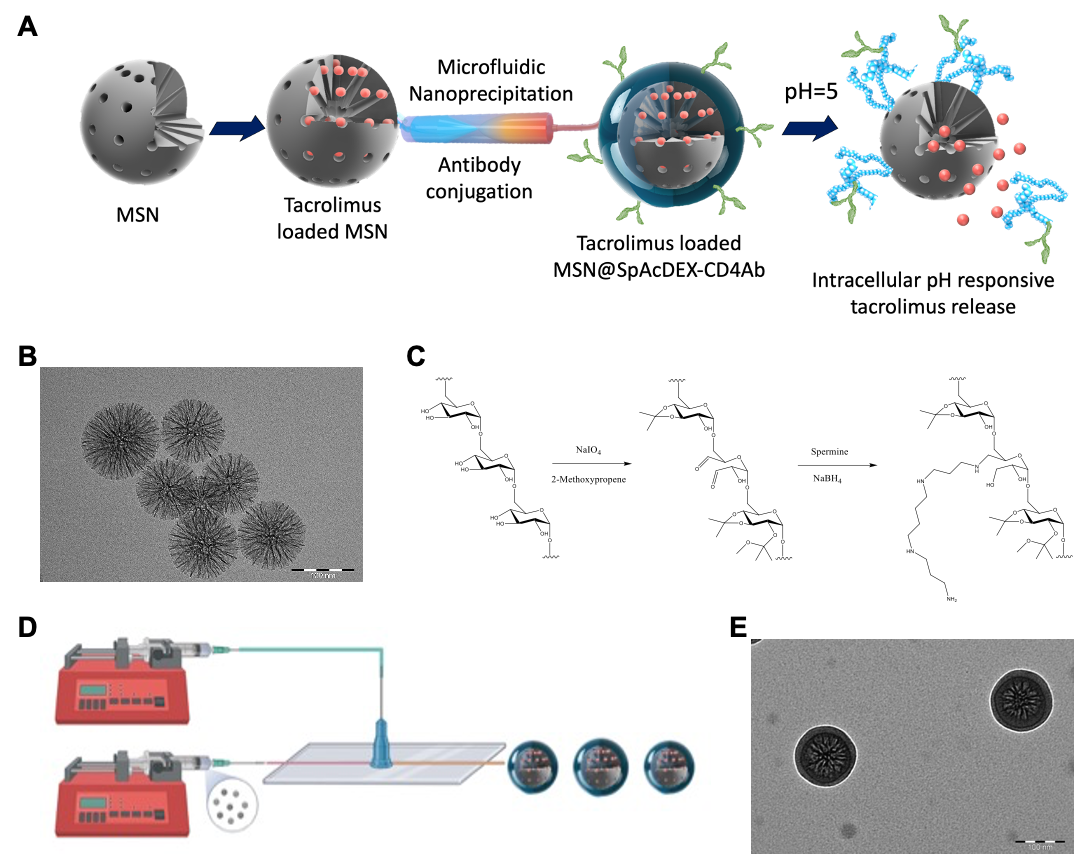


***Figure S2. The fabrication procedure of nanocomposites.*** *(A)Schematic representation of MSN@SpAcDEX-CD4Ab formation under microfluidics encapsulated by targeted antibody. (B) The structure of mesoporous silica nanoparticles (MSN, Bar = 100 nm). (C) Schematic representation of the synthesis procedure of spermine modified oxidized acetalated dextran (SpAcDEX). Spermine-Ac-DEX was prepared using reductive amination chemistry to conjugate spermine to Ac-DEX. (D) Microfluidics chip for nanocomplexes fabrication. The overall architecture of the chip. Sequentially, from left to right, the drug-loaded nanoparticle ethanol suspension is introduced into the side of the chip attached to the coverslip; the external phase aqueous solution is injected from the top inlet; and the nanocomplex is obtained at the interface where the two phases are miscible. (E) The TEM image of SpAcDEX coated MSN after microfluidics (MSN@SpAcDEX, Bar = 100 nm).*

**
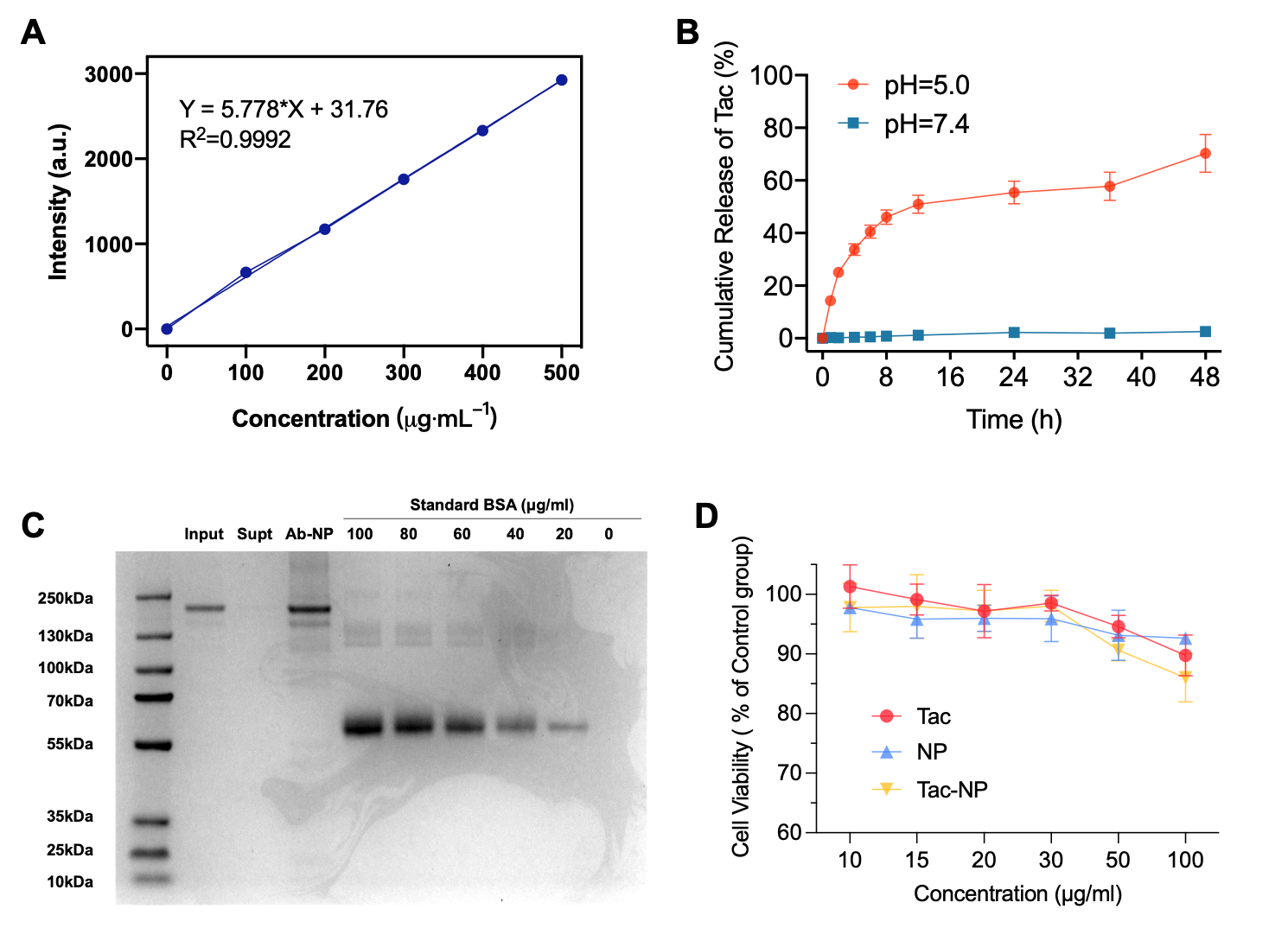
**

***Figure S3. Characterization and biosafety assay of nanocomplexes.*** *(A) The standard curve of Tacrolimus in ethanol measured with HPLC. (B) The drug release study. Tacrolimus release profiles of mesoporous silica nanoparticles in acidic (pH = 5.0) and neutral (pH = 7.4) buffer at 37°C (mean ± SD, n = 4).* *(C) Antibody binding efficiency. Protein concentrations in the original CD4 antibody (Ab input), the supernatant (Supt) and precipitation (Ab-NP) after binding with nanocomplexes were visualized using SDS polyacrylamide gel electrophoresis and Coomassie blue dye staining and semi-qualified by comparing with standard bovine serum albumin (BSA) samples. (D) The viabilities of primary splenocytes after 12 h incubation with Tacrolimus (Tac), MSN@SpAcDEX (NP), and Tac@MSN@SpAcDEX (Tac-NP) were measured using WST-1 assay. Cell viabilities are represented as percentage (%) of the value in RPMI 1640 medium control group (mean ± SD, n = 4). Two-Way ANOVA with Turkey multiple comparisons found no significant difference in cell viabilities between groups.*

**
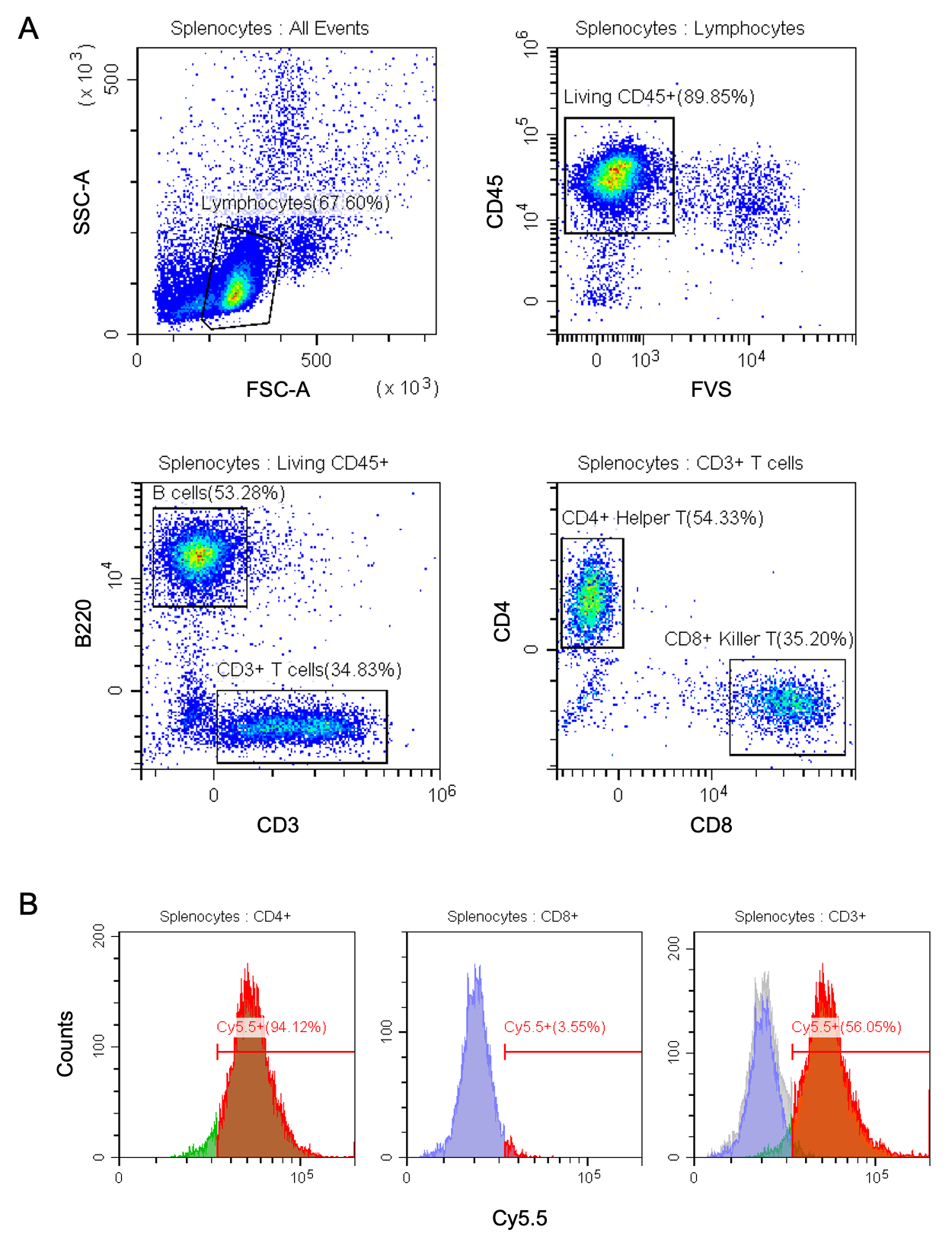
**

***Figure S4. Gating strategy for nanocomplex targeting detection.*** *The single splenocyte suspension was stained with Fixable Viability Stain (FVS)-440UV and anti-mouse CD45-BV786, CD3-PE-Cyanine5, B220-PE-Cy7, CD4-FITC, and CD8-BV605 antibodies. (A) Lymphocytes were determined using FSC and SSC signals. FVS^-^CD45^+^ gate was used to select living CD45^+^ cells. CD3^+^B220^-^ gate was used to select T cells. CD4^+^CD8^-^ and CD4^-^CD8^+^ gates were set for helper T cells and killer T cells, respectively. (B) Cy5.5 signal densities were detected in the cell populations of helper T cells (CD4^+^), killer T (CD8^+^), and T (CD3^+^) cells.*


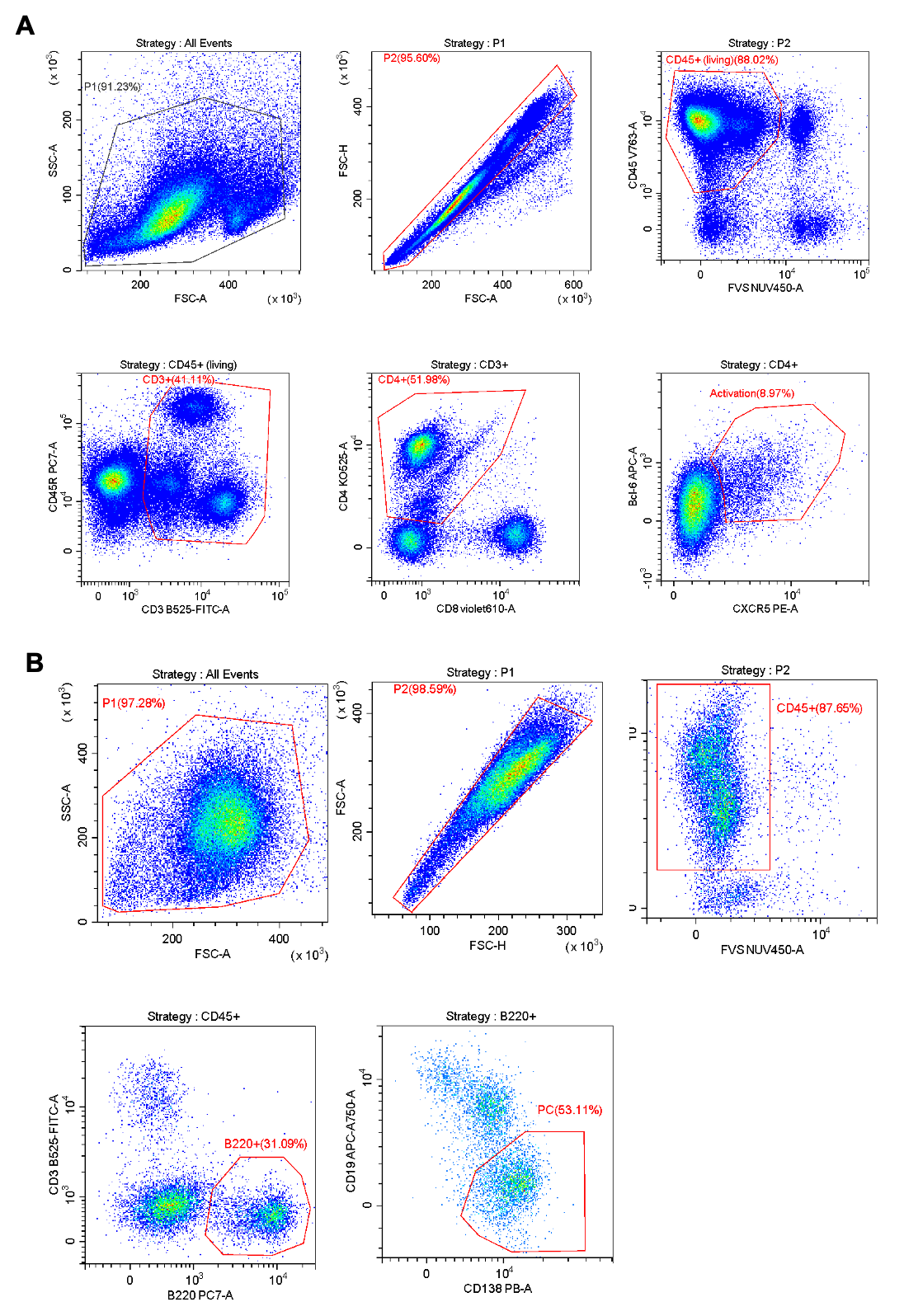


***Figure S5. Gating strategy for T follicular helper cells (Tfh) activation in spleen and plasma cell population for PBMC detection.*** *Splenocytes were stained with FVS-440UV, anti-mouse CD45-BV786, CD3-FITC, CD4-V500, CD8-BV605, CXCR5-PE, and Bcl-6-Alexa Fluor 647 antibodies. Lysing peripheral blood cells (PBMC) were stained with FVS-440UV and anti-mouse CD45-BV786, CD3-FITC, B220-PE-Cy7, CD19-APC-cy7, and CD138-BV421 antibodies. Single leukocytes were gated by SSC area (SSC-A), FSC area (FSC-A), and FSC area height (FSC-H). FVS^-^CD45^+^ gate was used to select living CD45^+^ cells. (A) CD3^+^B220^-^ cells were used to determine T cells and CD4^+^CD8^-^ helper T cells were selected. CXCR5^+^Bcl^-^6^+^ cells were the activated helper T cells. (B) B220^int/+^CD3^-^ gated the B cell population whereas CD138^int/+^CD19^-/int^ cells gated the plasma cells.*

***Figure S6. Body weight gain during 4-week drug administration.*** *Eight-week-old male C57BL/6 mice were treated with Saline, Tacorolimus (Tac), Tac@MSN@SpAcDEX (Tac-NP), Tac-NP-CD4Ab every two days for four weeks. The equivalent Tacrolimus dose was 1 mg/kg. Body weight (gram, g) of all mice was measured weekly. Data are shown as mean ± SD. Two-Way ANOVA with Turkey multiple comparisons showed no significant difference between groups.*


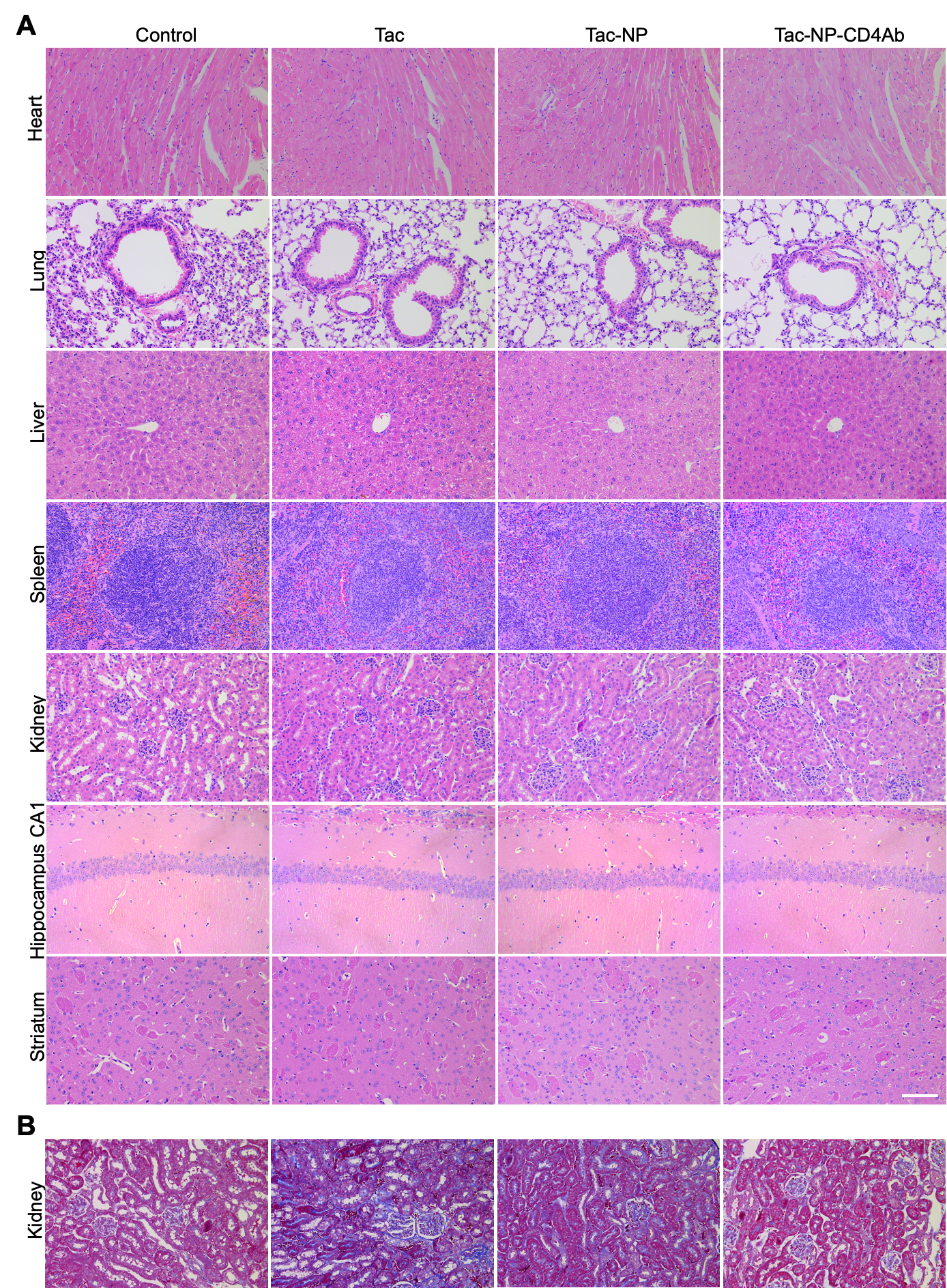


***Figure S7. Histological H&E staining of main organs and Masson’s trichrome staining of Kidneys.*** *Eight-week-old male C57BL/6 mice were treated with Saline, Tacorolimus (Tac), Tac@MSN@SpAcDEX (Tac-NP) and Tac-NP-CD4Ab every two days for four weeks. The equivalent Tacrolimus dose was 1 mg/kg. (A) Paraffin sections of heart, lung, liver, spleen, kidney, and brain (hippocampus CA1 and striatum) organs were stained with haematoxylin-eosin (H&E) staining (Bar = 100 μm). (B) Paraffin sections of kidney were stained with Masson’s trichrome staining (Bar = 100 μm).*
